# Supplementary figures and images for: The global population stru cture of Lacticaseibacillus rhamnosus and its application to an investigation of a rare case of infective endocarditis
Source: PLoS One. 2024 Aug 30;19(8):e0300843. doi: 10.1371/journal.pone.0300843 (PMC11364288; doi:10.1371/journal.pone.0300843)

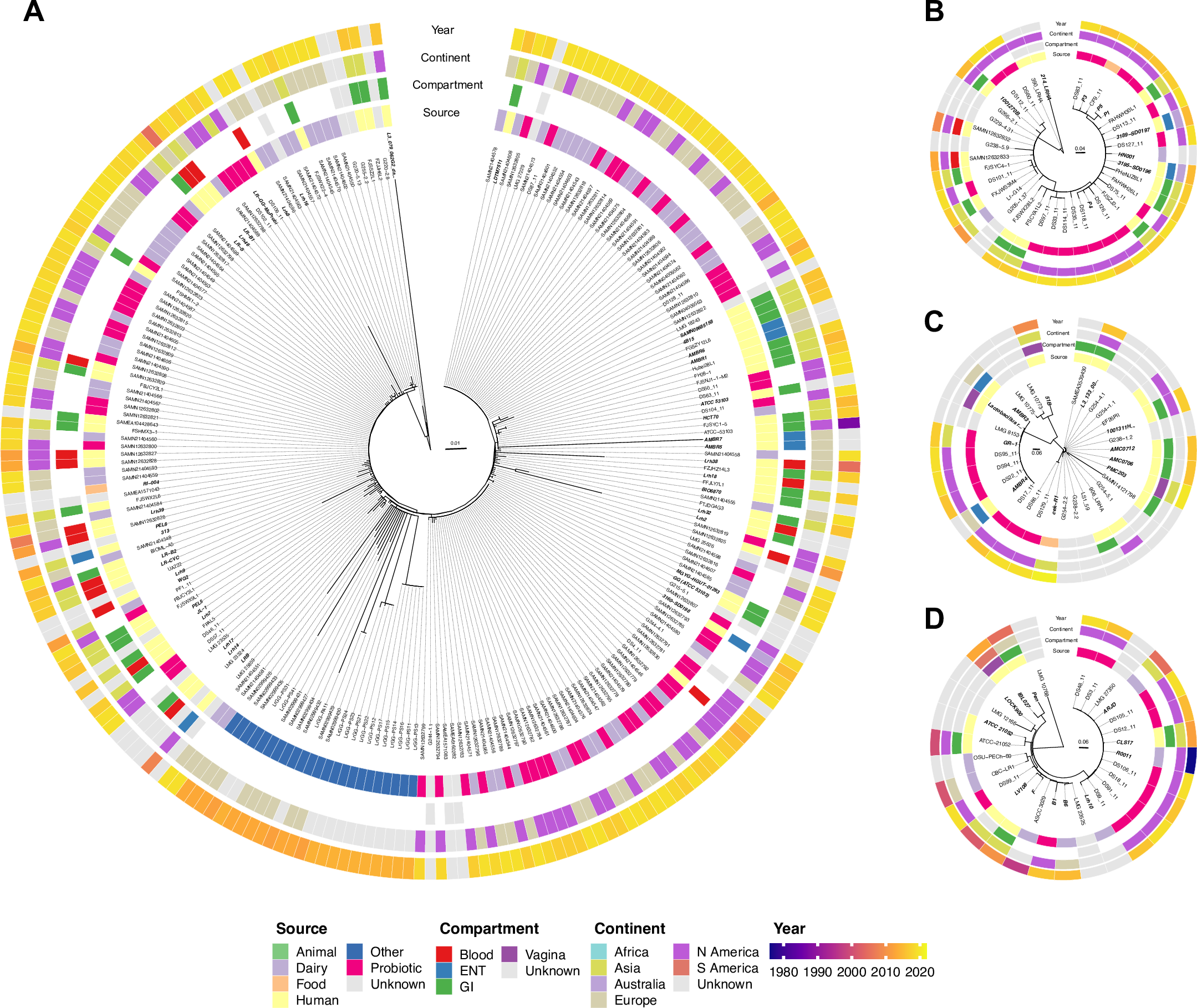

Supplement: S1 Fig — Maximum likelihood phylogenetic trees from whole genome alignments of sequences from clades GG (A), C1 (B), C2 (C), and C3 (D) to the reference genome sequence of strain ATCC 11443. Rings from inside to outside indicate, in order: 1. isolation source, 2. body site of isolation for human sourced isolates (compartment), 3. continent of isolation, and 4. year of isolation. Taxon labels in bold indicate sequences for which only genome assemblies were available. Scale bar indicates genetic distance. (TIF) [file pone.0300843.s001.tif]

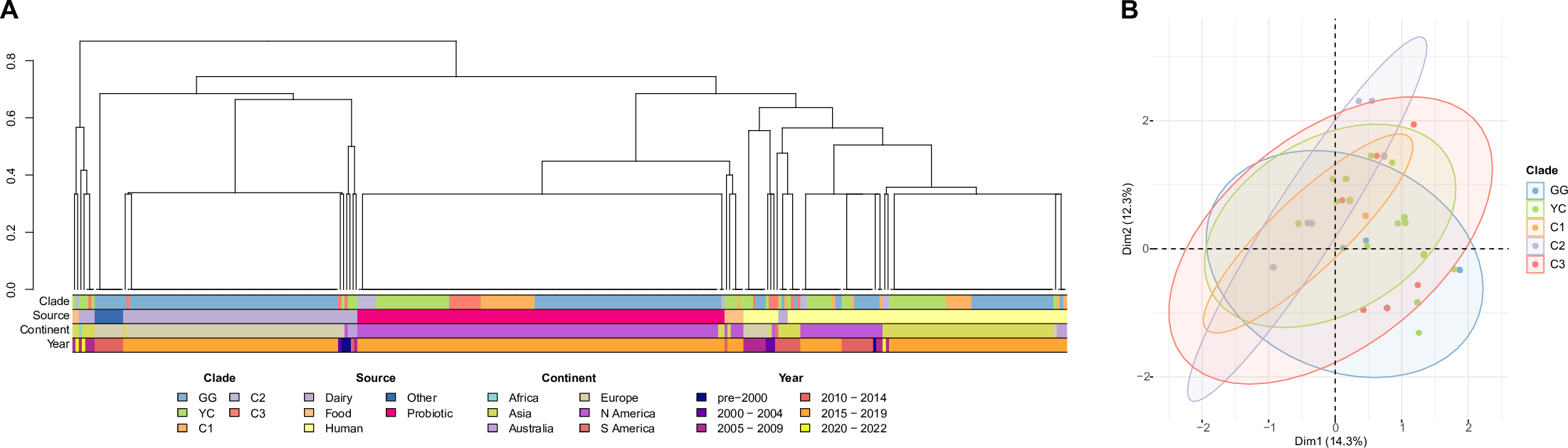

Supplement: S2 Fig — Only demographics from sequences with no missing values for source, continent, or year were used (n = 314). (A) Hierarchical clustering results using average linkage based on Gower distances among demographic characteristics Source, Continent, and Year. (B) Multiple component analysis of demographic characteristics with individuals colored by Clade membership. Ellipses indicate concentrations of individuals based on normal distribution. (TIF) [file pone.0300843.s002.tif]

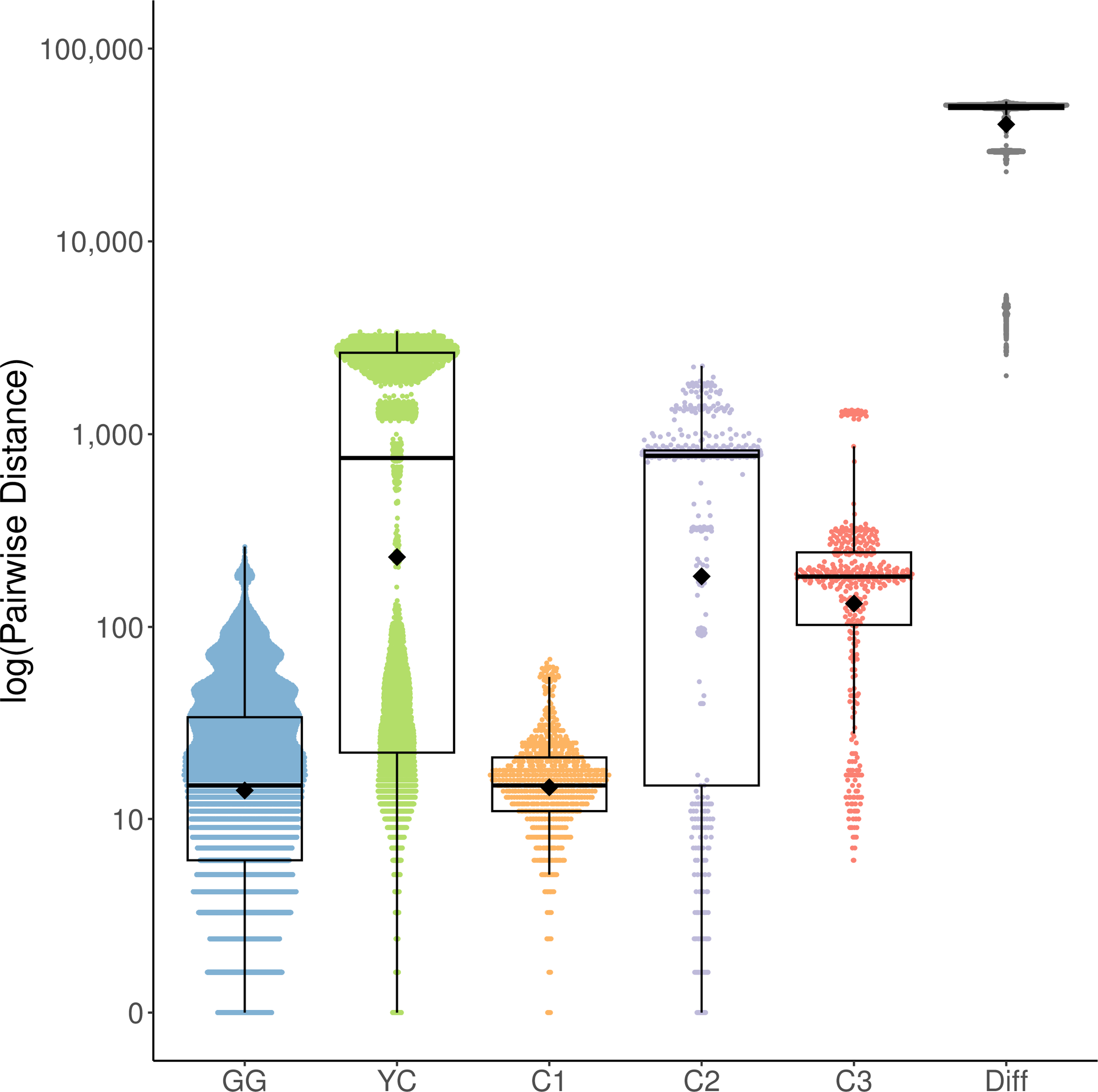

Supplement: S3 Fig — Dots indicate all pairwise genetic distances as measured by single nucleotide variant (SNV) differences among the 230 sequences in clade GG, 177 clade YC sequences, 39 clade C1 sequences, 31 clade C2 sequences, 29 clade C3 sequences or among comparisons between all pairs of sequences across the five clades (“Diff”). Overlying box and whisker plots indicate median SNV counts (thick black line) as well as first and third quartiles. Whiskers indicate 1.5 x the interquartile range (IQR) and average SNV counts per group are indicated by black diamonds. Y-axis is plotted on the log10 scale. (TIF) [file pone.0300843.s003.tif]
