# Supplementary material for: The global population stru cture of Lacticaseibacillus rhamnosus and its application to an investigation of a rare case of infective endocarditis
Source: PLoS One. 2024 Aug 30;19(8):e0300843. doi: 10.1371/journal.pone.0300843 (PMC11364288; doi:10.1371/journal.pone.0300843)
Supplement: Attachment — Submitted filename: Response to Reviewers.docx [file pone.0300843.s007.docx]

Reviewer #1: Comments and Suggestions for Authors

The manuscript examine the population structure of Lacticaseibacillus rhamnosus and investigate the utility of using bacterial genomics to identify the source of invasive Lacticaseibacillus infections. Although the paper is well written and structured and the experiments are well planned, some questions need to be explained or clarified, as indicated as follows:

INTRODUCTION

1. " formerly Lactobacillus" appears in the Line 4, which should be supported by references.

The manuscript describing the reclassification of the genus *Lactobacillus* into 25 genera including *Lacticaseibacillus rhamnosus* (Zheng J et al, International Journal of Systematic and Evolutionary Microbiology. 15 April 2020 70(4)) has now been referenced on page 4, line 88.

2. The introduction has no logic, please rearrange it.

We have revised the Introduction to better highlight the background literature supporting the study.

1. Why should we study the relationship between Lacticaseibacillus rhamnosus and endocarditis, or what is the necessity, please revise and add.

Although *Lacticaseibacillus rhamnosus* is a common component of or additive to foods and probiotic products and several strains are considered Generally Recognized As Safe (GRAS) by the United States Food and Drug Administration (FDA) (<https://www.cfsanappsexternal.fda.gov/scripts/fdcc/?set=GRASNotices&sort=GRN_No&order=DESC&startrow=1&type=basic&search=rhamnosus>), there have nevertheless been several reports of severe invasive infections including endocarditis caused by *L. rhamnosus.* We have referenced reports of some of these cases in the Introduction*.* Whether these infections are more likely to be caused by commensal *L. rhamnosus* strains or by *L. rhamnosus* strains ingested as additives to food or probiotic supplements is critical to their prevention. We have revised this section to better highlight justifications for the study.

2 in the introduction. Author thought WGS is not accurate enough, why should this method be used for your research?

We thank the reviewer for highlighting this confusingly worded section of the Introduction. The point we were attempting to make was as follows: WGS approaches have been effective for suggesting origins and tracking transmissions in many infectious diseases and are increasingly used to investigate infections caused by many viruses, bacteria, fungi, and parasites. Thus, we chose to apply a WGS approach to investigate a case of *L. rhamnosus* endocarditis in our hospital. However, we found that the lack of strain-to-strain diversity among publicly-available genome sequences of *L. rhamnosus* limited the utility of WGS to distinguish between food additive, commensal, and invasive strains of this organism. Our point, therefore, is that this commonly used approach is not universally applicable and fails in the case of *L. rhamnosus.* We have revised this section to try to better highlight our rationale.

3. At the end of the introduction, the author believes that WGS cannot identify the real source of bacteria. What is the purpose of the research after that?

Before we began the study we anticipated that WGS might elucidate the source of our patient’s bacteremia; However, we discovered that the clonality of many human-associated and commercial isolates limited our ability to definitively identify whether recent yogurt consumption may have led to his illness. Without a thorough analysis of the global population structure of the species based on publicly available sequences as well as sequences of isolates obtained directly from store-bought yogurt samples, we would have been unable to recognize the highly clonal nature of this organism. At its conclusion, our study suggests that despite its high resolution, WGS may still fall short in tracing the origins of infections when applied to certain rare pathogens with highly clonal population structures and/or dominated by conserved type strains heavily used in commercial applications such as *L. rhamnosus*. This was not our expectation before embarking on the study and we came to this conclusion only after undertaking the study. We have revised the Introduction to try to clarify this.

RESULT

1. Please explain whether the different branches of Lactobacillus rhamnosus are related to the source.

A conclusion of our study is that the different branches of the phylogenetic tree do NOT segregate by source. The five largest clades in our study, including GG and YC, each contained representatives isolated from dairy, food, human, and probiotic sources, as shown in Figure 1B, Supplemental Figure 1, and quantified in the newly-added Figure 2. We have also now performed an assessment of relationships between source of isolation and phylogenetic clade as shown in the new Supplemental Figure 2.

2. The result has no logic, please rearrange it. For example, to supplement the relevant information of the genome, the relationship between different branches and isolates.

We have now rewritten the Results section to better explain the rationale for each analysis and to present the flow of our approach as we proceeded through the investigation.

DISCUSSION

1. Please analyze the discussion section in conjunction with other references and do not continue to describe the content of the results.

We thank the reviewer for this suggestion. We have edited the Discussion section to remove a paragraph that contained a redundant restatement of the results.

2. Lacticaseibacillus rhamnosus was detected in the blood of the patient. The possible cause is not discussed, please add.

Most bloodstream infections caused by *Lactobacillus* and other gut flora organisms are presumed by clinicians to have arisen due to gut translocation. Thus, it is assumed that the source of this patient’s bacteremia was his GI tract. We have revised the manuscript to clearly state the two possible source populations of *L. rhamnosus* in this patient’s GI tract: (1) the *L. rhamnosus* originating from his regularly ingested yogurt, or (2) the commensal *L. rhamnosus* inhabiting his GI tract. We have revised this section to indicate that the source of the infection was not able to be definitively established in this patient but that it was suspected to be one of these two reservoirs of bacteria.

3. The relationship between Lacticaseibacillus rhamnosus and geography and origin is not clearly discussed in this paper. In addition, Lacticaseibacillus rhamnosus in blood may come from food, which is not discussed in detail in this paper. The authors may refer to other relevant studies on the genome of L. rhamnosus, such as A large-scale comparative genomics study reveals niche-driven and within-sample intra-species functional diversification in Lacticaseibacillus rhamnosus(doi.org/10.1016/j.foodres.2023.113446)

We thank the reviewer for this comment. We have now expanded upon our analysis of the population structure of *L. rhamnosus* by identifying and investigating the major phylogenetic clades in our population. Clustering based on phylogenetic tree branch length identified five separate clades of sequencing comprising a total of 70% of the study population. Using this approach did not result in a difference in the number of sequences in the GG clade, but expanded the size of the YC clade from 79 to 117 sequences through the inclusion of some phylogenetically more distant, but still closely-related sequences. We have added a new Figure 2 to compare proportions of sequences in each of the five major clades for each of the available origin, geography, and temporality characteristics. Furthermore, we have now included Supplemental Figure 2 that explores relationships between these characteristics and the phylogenetic clades using both hierarchical clustering and multiple component analysis. Based on these analyses we conclude that other than a predominance of GG clade sequences among dairy isolates from Europe seen in the hierarchical clustering analysis, there is no clear relationship between the phylogenetic clades and origin, geography, or date of isolation. These results concur with the findings in the recent study cited above, which was published after our manuscript was first submitted. We have now referenced this study in our discussion. Finally, we have also added that a limitation of our study and these analyses is that the sequences and data included came from multiple separate studies and publicly available data and so could be biased by separate study goals, collection strategies, and decisions to deposit (or not deposit) data in the public database for inclusion here.

References

1. Please unify the format

We have revised the bibliography to remove redundant text and are using the PLoS EndNote Output Style for consistency in the reference and bibliography styles.

Reviewer #2:

TITLE: It is suggested that the authors revise the title and make it more concise and appealing.

We have now modified the title to be more descriptive of the study approach: “The global population structure of *Lacticaseibacillus rhamnosus* and its application to the investigation of a rare case of infective endocarditis.*”*

ABSTRACT

In background, Correct to formally knowns as

We thank the reviewer for this suggestion, however in our reading publications describing revisions in taxonomy generally use “formerly” rather than “formerly known as.” Examples in the Lacticaseibacillus literature include <https://pubmed.ncbi.nlm.nih.gov/34373437/>, <https://pubmed.ncbi.nlm.nih.gov/37396874/>, <https://pubmed.ncbi.nlm.nih.gov/36207675/>, among others. For consistency with the prior literature and in pursuit of economy of language in the abstract, we have elected to maintain the current language.

Some rephrasing is needed.

What do you mean by population structure?

We apologize for any ambiguity in our usage of this term. Population structure here refers to the overall genetic diversity of the population of *L. rhamnosus* represented by the available genome sequences in the global public database. We have updated the Lay Summary to include this description.

LAY SUMMARY

What is meant by the population structure

See above comment.

INTRODUCTION

In first line correct to ‘and to prevent…’

Added.

Insert reference for the statement ‘a common human and animal commensal species…’

We have added a reference discussing commensalism among *Lactobacillus* species: reference #3 in the bibliography, Xiao Y et al. Gut Colonization Mechanisms of *Lactobacillus* and *Bifidobacterium*: An Argument for Personalized Designs. Annu Rev Food Sci Technol. 2021;12:213-33.

Specify which type of commercial products.

We have expanded this section to include examples of commercial products that include *Lactobacillus* species.

What do the authors mean by saying ‘For this reason…’?

This statement has been removed.

What is meant by What do you mean by “escape from GIT” Clarify.

We have included the clarification that escaping the GI tract means to enter the surrounding tissues and/or the bloodstream resulting in invasive infections.

Cite reference for the statements where ingestion of lactobacillus containing foods poses a risk for patients and sick individuals.

We have included several references to this risk earlier in the paragraph where we describe bloodstream infections and endocarditis (references 5 – 8), among which the patients in the described cases were largely immunocompromised.

The use of the term strain ought to be used instead of isolates

We appreciate that opinions differ in the field on the use of these terms but feel that the use of the term “isolate” is best in this situation. We are following nomenclature guidelines laid out in the following manuscript: A. van Belkum et al, “Guidelines for the validation and application of typing methods for use in bacterial epidemiology”, Clinical Microbiology and Infection, 2007, 13(3):1-46, <https://doi.org/10.1111/j.1469-0691.2007.01786.x>. In the context of our report we largely describe genetic and clinical features of individual isolates cultured from clinical or other specimens which may or may not share genetic similarity with certain defined strains: *L. rhamnosus* GG (LGG), for example. But as this study almost exclusively describes and analyzes bacteria cultured from individual patients or other specimens and not necessarily from laboratory strains or type strain repositories, we feel that using the term “isolate” is more appropriate in these cases.

The third paragraph of the introduction section carries statements which are more suited for results and conclusion sections, for example ‘We demonstrate that food, probiotic, GI commensal, and

infection isolates are intermixed across the phylogenetic tree and frequently show surprisingly

little strain-to-strain sequence variability. These findings suggest that WGS may not be useful in

determining the source of L. rhamnosus infections….’ Similarly, the last 4 lines of the same para are also repetitive and belong to the conclusion section and not the introduction part of the article.

We sought to structure our Introduction section in a standard research article style by providing some background and justification for the study and ending with a brief summary of the main results and conclusions of our investigations. We agree, however, that the final paragraph of the introduction was overly detailed with regards to study results. We have revised and rearranged the introduction to better justify the study and lead into the Results section.

Rephrase statement given against cited reference 10

We have restructured this sentence which in the revised manuscript now refers to reference 12 (Agrawal S et al.) on page 5, starting on line 114.

RESULTS

The authors must give a clear conceptual explanation of the term’s unique whole genome assemblies and Illumina read sets in the context of their rationale of doing this work and experimental design

can we conclude from the sequencing data if there is significant variability in the data (supplementary table 1) such as # of read pairs/bases, alignment coverage and genome size? Or even missing for many strains... explain why this is not the case here!

Again, great variation in sequencing data provided in supplementary table 1. So, is it justifiable to interpret only 10 or 11 SNVs difference? The total number of bases were provided only for DSM. Why is it so?

We sought to include as many publicly-available whole genome sequences as possible to try to best assess the genetic diversity of the species associated with human- and non-human sources. To incorporate as much data as possible we used two types of WGS data available in the public database: whole-genome assemblies and unassembled sequencing read sets generated from WGS. The missing sequencing data in Supplemental Table 1 reflects the data available for these two data types, which are identified in Column O: “Type”. As many sequence read sets do not have an associated whole genome assembly in the database, no assembly characteristics (i.e. assembly contigs, assembly size, N50, etc.) could be reported for these read sets. Similarly, for WGS data only available as assemblies, no data on read numbers or number of read bases exist in the database. As described in the manuscript, we identified instances where the same isolates had both types of data deposited (assemblies and reads) and excluded the assembly sequence to avoid data duplications. As described in the Methods, we also excluded potentially low-quality assembly and read sets using the criteria described which to increase the confidence of our alignment analyses within the limitations we have described above. We have now added a statement to the Results section to refer the reader to the description of our quality filtering strategy in the Methods section. Additionally, we now state that we identified unique isolate sequences as those having unique NCBI BioSample IDs.

The result section ends with a summary which is the main conclusion of your study? Was this not obvious already?

We have now removed this summary.

DISCUSSION

Explain the rationale ‘of combining WGS and assemblies…’

We apologize for the lack of clarity in this statement. We have now updated this section to explain that variant-calling software written to analyze data from sequencing read alignments differ in their methodologies from variant-calling software designed to analyze alignments of whole genome assemblies. We now state that we sought to reduce bias in our analyses by generating pseudoreads from the assembly data, which allowed us to use the same analysis approach for both data types.

MATERIAL & METHODS

In the section of public sequences, the manner of excluding assembly sequences appears arbitrary, contig counts or total genome size limits are vague, must be clarified, and restated.

We have now revised this section to improve its clarity and include our justification for selecting these exclusion criteria. We selected these criteria to try to balance including genome sequences with sufficient quality to conduct a broad assessment of overall population structure of the species while excluding the lowest quality sequences most likely to cause grossly inaccurate results in the phylogenetic analyses.

Clarify how ‘read sets were further filtered to randomly select one set of reads from multiples…’?

We have rewritten this section to better describe cases where multiple read sets representing repeat sequencing of the same isolate (represented by a single BioSample ID) were handled to select one of the available read sets. A supplemental table of excluded read sets is now provided separately (Supplemental Table 2).

How do you compare the quality of the 419 read sets with WGS? What are the benchmarks? It would be giving this comparison in a tabulated form

no detail of illumina read sequences has been provided. What is the quality of reads? Is it comparable? For example, we cannot compare 60% quality reads with 95-100%

We have now included more detail of the sequencing read sets included as well as the FastQC sequence quality assessment results. All read sets included passed the FastQC per-sequence quality filter threshold.

In the section Single nucleotide variant identification, ‘What is meant by SNV quality of scores of less than 200, selection of read consensuses, read depths?

The SNV quality score is a standard assessment calculated by bcftools and other read alignment software producing a Variant Call Format (vcf) output file. The quality score (“QUAL”) is an assessment of the probability that a variant (“ALT”) called by the software at an alignment position is incorrect. Per the vcf file format manual (<https://samtools.github.io/hts-specs/VCFv4.2.pdf>), the QUAL score is the “Phred-scaled quality score for the assertion made in ALT. i.e. −10log_10_prob(call in ALT is wrong ).” Read consensus is the percentage of reads aligned to a particular position that share the predicted base call. Read depth is the total number of reads aligned to a position. We have now revised the text to clarify this terminology.

In the section Phylogenetic analysis, what are separate complete sequence alignments. What is the basis of this consensus building?

By “separate complete sequence alignments” we intended to say that one core genome alignment was generated from all 606 genome sequences from variant positions with a base found in all 606 genomes, a second core genome alignment was generated from the 79 YC genomes from variant positions with a base found in all 79 of the YC genomes, and a third core genome alignment was generated from the 230 GG genomes from variant positions with a base found in all 230 GG genomes. We have added a description of how consensus sequences were produced from each alignment to the section above.

In the section Patient Consent Statement, the authors make some conflicting statements regarding patient consent, they state that patient consent is not possible because of death of the patient but in the same paragraph, they also claim that it is not a requirement.

Kindly provide reference or describe the exact rule which supports that the ‘the study is not considered human subject's research

We apologize if these statements appear to be conflicting. Per guidelines produced by the US Department of Health and Human Services Regulation 45 CFR 46, 2018 Requirements (2018 Common Rule), section 46.102 “Definitions for purposes of this policy”, human subject refers to a “living individual” (<https://www.hhs.gov/ohrp/regulations-and-policy/regulations/45-cfr-46/revised-common-rule-regulatory-text/index.html#46.102>). Per NIH guidelines based on these Regulations, “If all the specimens or data will come from cadavers or otherwise deceased individuals, your study is not considered human subjects research.” (<https://grants.nih.gov/policy/humansubjects/hs-decision.htm>). We have now revised the text to clarify this.
