# Supplementary material for: The global population stru cture of Lacticaseibacillus rhamnosus and its application to an investigation of a rare case of infective endocarditis
Source: PLoS One. 2024 Aug 30;19(8):e0300843. doi: 10.1371/journal.pone.0300843 (PMC11364288; doi:10.1371/journal.pone.0300843)
Supplement: Attachment — Submitted filename: Response to Reviewer 3.docx [file pone.0300843.s008.docx]

Reviewer #3: The article written is important for the area of probiotics and the conclusions obtained may generate controversy, mainly because the text establishes a direct relationship between the presence of the Lactobacillus species in the patient's bacteremia and in yogurts that are of mass public consumption. Therefore, it is important to carry out an exhaustive review of the text and of the statements that are being used. In addition to this, it is important to make the following changes:

1. Replace the terminology "flora" (in disuse) by microbiota.

We thank the reviewer for highlighting the update in terminology. All instances of “flora” in the manuscript have been replaced with “microbiota”.

2. Include more bibliographic references in lines 56, 86-90, 92, 274-275 (original manuscript) and in all the sentences that are convenient to better support the statements that are made

We have added more supporting references throughout the manuscript as requested. These include:

Line 84, references 5-8

Line 89, references 13-14

Line 91, references 15-17

Line 94, reference 19-20

Line 293, reference 27

3. Restructure the results to make them more understandable, including subtitles to separate the various analyses performed.

Subtitles have been added to the Results section to better separate the study findings and some sections have been rearranged to try to improve readability and flow.

4. Indicate whether the yogurt brands analyzed include the one that the patient consumed (based on the relatives' statement) and justify why these brands were chosen (were they randomly selected? How can one justify the selection of 4 yogurt brands as a significant sample of all the yogurt brands offered on the market?)

During the course of his illness the patient had shared with clinicians that he would eat yogurt frequently to improve his gut health but was not asked at the time which brand or brands he was consuming. During the critical phase of his illness when he was no longer able to answer questions, his family was asked about his yogurt preferences. They were not able to definitively recall his preferences, but named one brand as a yogurt brand that the patient would frequently consume. They could not state with confidence that this brand was exclusively consumed by the patient, nor whether he consumed it more frequently than other brands. This brand was included in our study and is anonymized as “Brand A” in the report. The other three brands purchased were a convenience sample selected from among other brands available in the supermarket at the same time that “Brand A” was purchased. These four brands are representative of nationally-available yogurt brands that are also broadly available from supermarkets in our region of the U.S. We have updated the manuscript to better explain the rationale for the yogurt brand studied (line 214-217).

5. In the abstract the "core genome" is mentioned, but nothing else is indicated in the rest of the paper. Justify why the analysis was done only on the basis of the core genome, how it was defined and what the core genome comprises.

We thank the reviewer for pointing out the lack of clarity on this portion of the analysis. We have updated the “Phylogenetic analysis” section in the Methods to more clearly highlight how the core genome alignments were produced for phylogenetic analysis (Line 446).

6. In the discussion, cite other studies that have performed similar analyses or indicate that this is the first one.

We have revised and updated the first paragraph of the Discussion section to highlight the novelty of our study. Briefly, there have been few published reports of the population structure of *L. rhamnosus* and only one (Nadkarni et al, BMC Genomics 2020) that directly examined the genomic context of clinical *L. rhamnosus* isolates relative to the larger population structure. This prior study did not include any isolates associated with invasive bloodstream infection and found that the five clinical isolates studied were phylogenetically distinct from non-human source isolates. Our report is the first study to examine the phylogenetic context of a bloodstream invasive isolate and directly show that it is related to an isolate from a commercial yogurt source. It is also the first study we are aware of that describes the YC clade of globally distributed human- and non-human-associated isolates closely related to the Hansen 1968 type strain.

7. And finally, clarify that the condition of the patient from whom the strain was obtained was very critical and his decompensation is due to several factors. Also clarify that more analyses like this one (and even with greater statistical support) are needed to affirm that there is a relationship between probiotic consumption and the generation of serious infections.

We thank the reviewer for these suggestions.

With regards to the first comment, while the patient’s underlying conditions, including his age, history of prosthetic heart valve replacement, IgG monoclonal gammopathy, and his history of liver cirrhosis may have collectively predisposed him to a more severe outcome from infection, the patient’s decompensation and death was very likely directly attributable to the *L. rhamnosus* infection as indicated by his recurrent and prolonged bacteremia, the echocardiogram evidence of aortic valve vegetation, and his ultimate intracranial hematoma most likely caused by migration of infectious material from his infected heart valve to his brain. Given that a more detailed report of the patient’s case was previously published (Agrawal S et al, BMJ Case Rep. 2020), we present an abbreviated summary of the case in this manuscript and refer to the comprehensive previous report for more details on the patient’s history and condition.

To the second comment, we have added this as a potential limitation to the Discussion section (line 367).
